# Development of a nomogram model for predicting dementia risk in the older adult population of Weifang, Shandong Province, China: based on the biopsychosocial model

**DOI:** 10.3389/fpubh.2025.1499820

**Published:** 2025-02-21

**Authors:** Pengxin Geng, Wenjia Feng, Weiqin Cai, Hongqing An, Anning Ma

**Affiliations:** ^1^School of Public Health, Shandong Second Medical University, Weifang, China; ^2^School of Management, Shandong Second Medical University, Weifang, China

**Keywords:** dementia, influencing factors, nomogram, China, prediction

## Abstract

**Background:**

Dementia has emerged as a predominant health challenge. However, there is a notable research gap in the collective screening of dementia risks. Hence, there is a pressing need to formulate a dementia prediction tool tailored to the older adult demographic, enabling the identification of high-risk individuals for dementia.

**Methods:**

From May to October 2023, a multi-stage sampling method was utilized to survey older adult individuals aged 60 and above in Weifang. This study employed the Brief Community Screening Instrument for Dementia (BCSI-D) for the identification of individuals with dementia. We integrated the biopsychosocial model to construct a comprehensive pool of factors influencing dementia. Employing the least absolute shrinkage and selection operator and multivariate logistic regression analyses, independent influencing factors were identified to construct a nomogram prediction model.

**Results:**

Six hundred and sixty valid questionnaires were included in the final analysis, with a validity rate of 95.23%. We identified 178 cases of dementia using the BCSI-D. Napping, lack of concentration, self-assessed health status, education level, residence, social interaction and medical insurance were independent influencing factors for dementia. The efficiency analysis of the prediction model, constructed using these factors, demonstrated area under the receiver operating characteristic of 0.751 for the training set and 0.794 for the test set. The decision curve analysis threshold probabilities for the training and test sets were 5–60% and 1–60%, respectively. The calibration curves of both datasets exhibited a high degree of fitting with the predicted curve.

**Conclusion:**

We developed a dementia risk identification model with noteworthy predictive performance. The proposed model offers theoretical and data support for collective dementia screening.

## 1 Introduction

Dementia, as a prevailing neurodegenerative condition among the older adult, has emerged as a key global health concern (1). According to a dementia survey conducted by the World Health Organization, over 55 million individuals worldwide have dementia, with more than 60% of cases located in low-and middle-income countries (2). With the escalating trend of aging in China (3), the incidence of dementia in individuals aged 60 years or older has risen to 6.0% in the past 5 years. Projections suggest that by 2030, China will bear a substantial burden of dementia, with an estimated 22.2 million cases among the older adult (4, 5).

The onset of dementia is influenced by a multitude of factors. Genetic elements play a pivotal role. For example, the polymorphism of the apolipoprotein E (APOE) gene stands out as a primary genetic risk factor for late-onset dementia (6). Carriers of the APOEε4 allele could experience earlier amyloid pathology in the brain, heightening the risk of dementia, while the APOEε2 allele could potentially lower this risk (7). Beyond genetic factors, demographic factors such as female sex (8), rural residency (9), and lower education level (10) contribute to an increased vulnerability to dementia. Lifestyle choices also exert a significant impact on dementia onset. Studies indicate that maintaining a balanced diet (11), engaging in moderate exercise (12), and ensuring good sleep quality (13) can lower the risk of developing dementia. Contrastingly, adverse health conditions, including hypertension (14), diabetes (15), high cholesterol (16), depression (17), and poor self-rated health (18) amplify the risk of dementia. Social factors, such as active social engagement (19) and access to health insurance (20), are protective factors against dementia. Conversely, air pollution, traffic-related particulate matter, and other environmental pollutants exert harmful effects on the nervous system, elevating the risk of Alzheimer’s disease (21, 22).

Presently, there is no cure for dementia, while early risk identification primarily relies on laboratory tests (23), neuroimaging (24), and cerebrospinal fluid analysis (25). In recent years, several studies have turned their attention to using machine learning for predicting dementia risk. These studies often leverage clinical and biological data, such as images (MRI scans) (26), biomarkers (27, 28), and information extracted from MRI scans (29) for dementia risk identification. While clinical and biological data prove highly practical for individual dementia risk screening, they present drawbacks at the population level, including high costs and implementation challenges. Previous research has identified dementia risk factors as multifaceted and multidimensional (30). In comparison to clinical data, sociological data offer advantages such as greater breadth, increased accessibility, and lower cost, making them highly applicable in collective dementia risk screening. Therefore, it is crucial to adopt a population perspective and integrate sociological factors into research on dementia risk screening.

The biopsychosocial model (31) utilizes a sociological approach to explore the health domain, emphasizing the multi-level influences of psychosocial environments on human well-being. Applying the biopsychosocial model enables a systematic classification and comprehensive summary of risk factors for dementia, facilitating a nuanced identification of factors affecting dementia at various levels. This study predominantly adopted a collective perspective, employing the biopsychosocial model along with multidimensional factors encompassing biological, psychological, and social aspects. The aim was to construct a dementia risk prediction model specifically tailored to the older adult population in Weifang.

## 2 Materials and methods

### 2.1 Participants

A cross-sectional survey was conducted in Weifang, Shandong Province, China, from May to October 2023, using a multi-stage sampling method. Weifang is a typical coastal-inland hybrid city, incorporating both coastal and inland regions. To ensure the representativeness of the sample and minimize regional bias, Weifang was divided into three geographic regions: the coastal region (including Shouguang and Qingzhou cities), the inland region (including Weicheng, Hanting, and Fangzi districts), and the coastal-inland transition region (Zhucheng city). In the first stage, one county-level unit was randomly selected from each of the three geographic regions to ensure regional representation. In the second stage, three streets or townships were randomly selected within each chosen county-level unit as sub-sampling units. In the third stage, three communities were randomly selected within each selected street or township. Finally, older adult individuals aged 60 years and above were randomly recruited from each selected community as research subjects. Prior to the survey, permission was obtained from each community health service center. Before the onset of the survey, all investigators underwent standardized training. This survey was approved by the Ethics Committee of Shandong Second Medical University (Approval No. 2022YX057). All participants were informed about the purpose and content of the study before the survey and provided written informed consent. Inclusion criteria for the study were as follows: (1) aged 60 years and above; (2) residing in the area for more than 1 year; (3) willing and able to participate in the survey and provide informed consent. Exclusion criteria included: (1) conditions that hindered participation in the survey; (2) inability or refusal to provide informed consent. A total of 693 questionnaires were collected from 730 older adult participants, resulting in a response rate of 94.93%. After excluding 33 invalid questionnaires, 660 valid questionnaires were included in the final analysis, with a validity rate of 95.23%.

### 2.2 Definition of dementia indicators

Dementia was determined using the Brief Community Screening Instrument for Dementia (BCSI-D) for Dementia scale (32). This scale comprises both cognitive and informant sections. The cognitive section consists of seven questions, totaling nine items: (1) What is this called? (Interviewer pointing to the elbow); (2) What is the purpose of a hammer? (3) Where is the local market/supermarket? (4) What day of the week is it today? (5) What season is it currently? (6) Please point to the window first and then to the door; and (7) Can you remember the three things mentioned earlier? (ball, flag, tree). The informant section includes six questions: (1) Has there been a noticeable decline in your ability to handle tasks and communicate effectively? (2) Has there been a decline in your thinking and understanding abilities? (3) Do you experience frequent forgetfulness about the placement of belongings? (4) After a while, do you forget events from 2 days ago? (5) Do you sometimes forget your current location? (6) Do you experience difficulty with dressing?

In the cognitive section, correct answers scored 1 point, and incorrect answers are scored 0 points, resulting in a total score range of 0 to 9 points. A score of 4 or below indicates dementia, 7 to 9 points are considered normal, and 5 to 6 points suggest possible dementia. In the informant section, a response of “no” scores 1 point, while “yes” scores 0 points, resulting in a total score range of 0 to 6 points. For individuals with a potential dementia diagnosis, the informant section assists in confirming the presence of dementia (total score = cognitive section score – informant section score). The total score of the scale ranges from-6 to 9, with a score of 4 or below indicative of dementia. In this study, the Cronbach’s alpha value of 0.875 indicates good internal consistency and reliability of the questionnaire. The KMO value of 0.860 further confirms the questionnaire’s good construct validity.

### 2.3 The biopsychosocial model

The biopsychosocial model is a comprehensive medical framework used to analyze and comprehend health conditions and diseases. This model suggests that an individual’s health status is the outcome of the combined influences of biological, psychological, and social factors. In the biological dimension, variables include genetics, brain chemistry changes, neurodegeneration, and so on. The psychological dimension involves an individual’s mental health, emotions, personality, and cognitive functions. Social factors encompass an individual’s social interactions, cultural background, family, and work environment. Influencing factors for dementia were categorized based on the biopsychosocial model and previous studies (33, 34). Biological factors included variables such as sex, age, disabilities, brain injuries, visual impairments, hearing impairments, language function abnormalities, hypertension, hyperlipidemia, diabetes, tumors, chronic lung diseases, liver diseases, heart diseases, stroke, kidney diseases, arthritis, asthma, sleep duration, and napping. Psychological factors encompassed variables like worries, lack of concentration, low mood, difficulty in performing tasks, hopefulness, fear, poor sleep quality, happiness, loneliness, negative thinking, self-rated health status, emotional and mental issues, smoking, alcohol consumption. Social factors consisted of variables such as education level, marital status, place of residence, living alone, social interactions, exercise, physical examinations, health insurance, and community older adult care services. The selected variables in this study were based on previously identified dementia risk factors in the literature. Details of variable assignment is listed to Supplementary Table 1.

### 2.4 Statistical analysis

The data analysis was conducted using R software (version 4.3.1). To handle missing data, multiple imputation methods were employed (35). To ensure homogeneity between the training and validation sets, Chi-square (*χ*^2^) tests were performed on categorical variables. Descriptive statistics were reported using frequencies and percentages, while group comparisons for categorical variables were conducted using Chi-square tests.

Univariate analysis was performed using the Least Absolute Shrinkage and Selection Operator (LASSO) regression. LASSO is a linear regression method that incorporates regularization techniques, commonly used for data dimensionality reduction, feature selection, and constructing radiomics signatures. Compared to traditional linear regression models, LASSO excels in handling high-dimensional datasets and multicollinearity issues. By applying LASSO regularization, the method effectively reduces the data dimensionality and mitigates the risk of overfitting, making it particularly suitable for variable selection in small sample sizes. This makes LASSO a crucial tool in high-dimensional data analysis. During model development, 10-fold cross-validation was used to determine the optimal *λ* value. The complete dataset was then randomly split into a training set (*n* = 459) and a validation set (*n* = 201) in a 7:3 ratio (36).

All statistical tests were two-sided, with a significance level set at *p* ≤ 0.05.

### 2.5 Nomogram development

Variables with non-zero coefficients from the LASSO regression were included in the training set of the Logistic regression model. Factors with *p* < 0.05 in the Logistic regression model were considered as predictors. In addition, to assess the collinearity of variables in the Logistic regression, the variance inflation factor (VIF) was calculated (37). Finally, the model’s predictive performance was evaluated using the area under the curve (AUC), calibration curve, and decision curve analysis (DCA) curve.

## 3 Results

### 3.1 Dementia baseline analysis and homogeneity test

Following data screening, 660 samples were included. The non-dementia group comprised 482 individuals (73.03%), while the dementia group consisted of 178 individuals (26.97%). Among those with dementia, 96 were women (53.93%) and 82 were men (46.07%). Chi-square tests unveiled noteworthy correlations between dementia and arthritis (*χ*^2^ = 4.550, *p* = 0.033), sleep duration (*χ*^2^ = 6.859, *p* < 0.032), napping (*χ*^2^ = 9.179, *p* = 0.002), lack of concentration (*χ*^2^ = 8.041, *p* = 0.045), poor sleep quality (*χ*^2^ = 8.351, *p* = 0.039), self-rated health status (*χ*^2^ = 16.857, *p* < 0.001), education level (*χ*^2^ = 22.608, *p* < 0.001), place of residence (*χ*^2^ = 37.562, *p* < 0.001), social interactions (*χ*^2^ = 4.756, *p* = 0.029), physical examinations (*χ*^2^ = 5.107, *p* = 0.024), and health insurance (*χ*^2^ = 43.406, *p* < 0.001; Supplementary Table 2).

Upon setting the random seed at 123 for dataset partitioning, the training set comprised 459 cases, and the testing set comprised 201 cases. Within the training set, 128 cases (27.89%) were dementia cases, while in the testing set, there were 50 dementia cases (24.88%). Apart from heart disease (*χ*^2^ = 4.320, *p* = 0.038), maintaining hopefulness (*χ*^2^ = 9.679, p = 0.021), and smoking (*χ*^2^ = 5.868, *p* = 0.015), no significant differences were observed between the training and testing sets across the remaining 43 variables. This indicates homogeneity between the two sets in nearly all variables (Supplementary Table 3).

### 3.2 Identification of independent factors for dementia

**Figure 1:**
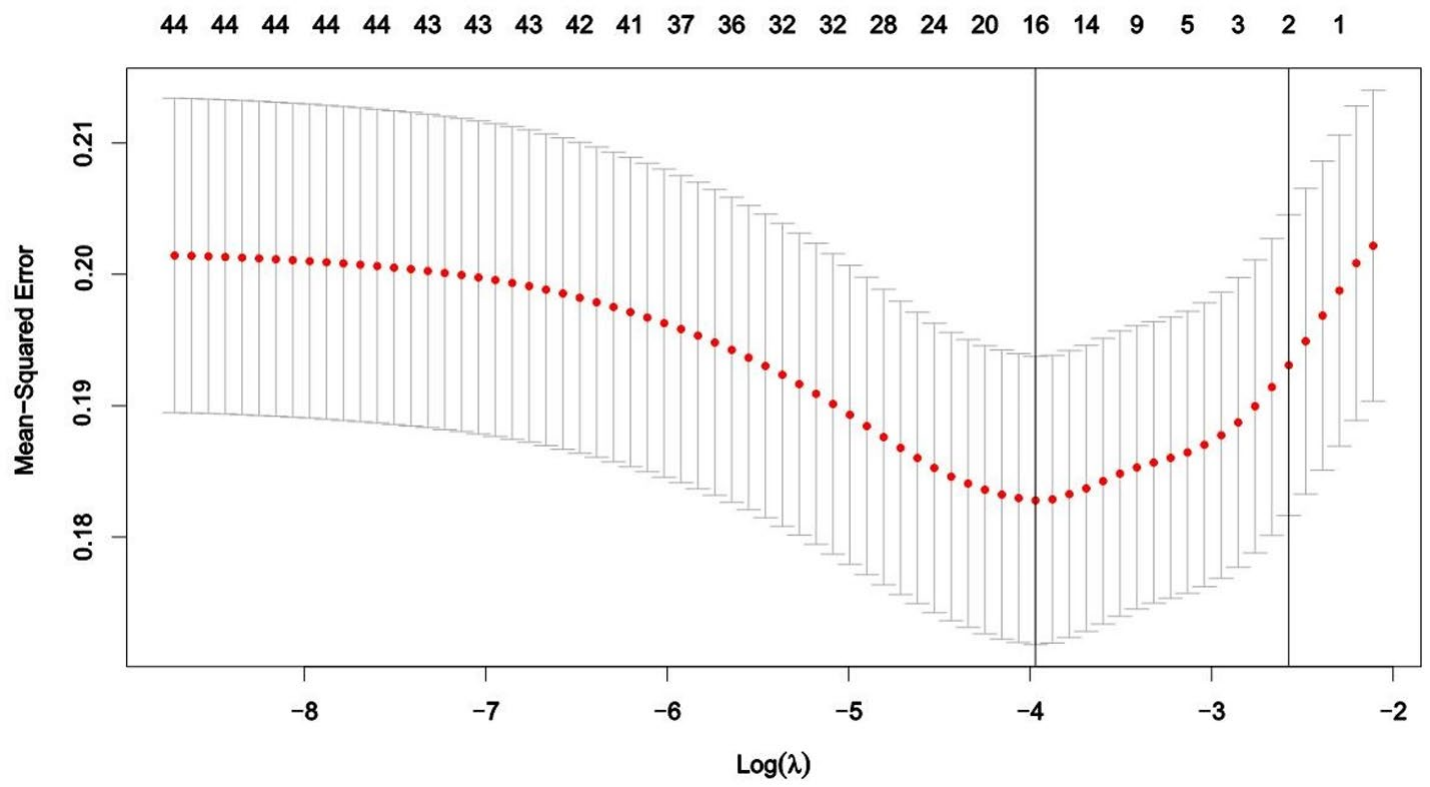
Feature variable selection using LASSO regression analysis.

Using LASSO regression analysis with 10-fold cross-validation, potential influencing factors for dementia were identified. As shown in Figure 1, the model identified 16 variables when the LASSO regression mean square error was minimized at *λ*.min = 0.019 and *λ*.1se = 0.076. These variables included gender (*β* = 0.047), physical disability (*β* = −0.036), hearing impairment (*β* = 0.087), hyperlipidemia (*β* = −0.054), heart disease (*β* = −0.001), sleep duration (*β* = −0.032), napping (*β* = −0.039), lack of concentration (*β* = −0.015), feeling hopeful (*β* = −0.003), self-rated health status (β = −0.042), education level (*β* = −0.040), place of residence (*β* = −0.062), social interaction (*β* = −0.021), moderate physical activity (*β* = −0.023), mild physical activity (*β* = 0.043), and health insurance (*β* = −0.196). Variables with non-zero coefficients were subsequently subjected to multiple logistic regression analysis. The results suggested that napping, lack of concentration, self-assessed health status, education level, residence, social interaction and medical insurance were identified as significant factors influencing dementia (Table 1). In addition, there were no multiple covariates between napping (VIF = 1.014), lack of concentration (VIF = 1.041), self-assessed health status (VIF = 1.022), education level (VIF = 1.054), residence (VIF = 1.060), social interaction (VIF = 1.006) and medical insurance (VIF = 1.015).

**Table 1:** Identification of independent influencing factors using multifactorial logistic regression.

Variable	OR	95% CI	*p*- value
Gender			
Male	1.000		
Female	1.280	0.857–1.917	0.857
Physical disability			
No	1.000		
Yes	0.495	0.107–1.687	0.302
Hearing impairment			
No	1.000		
Yes	1.821	0.821–3.950	0.132
Hyperlipidemia			
No	1.000		
Yes	0.650	0.261–1.485	0.326
Heart disease			
No	1.000		
Yes	0.547	0.221–1.246	0.168
Sleep duration			
≤5 h	1.000		
6 to 8 h	0.947	0.461–1.895	0.879
9 h	0.721	0.463–1.126	0.149
Napping			
No	1.000		
Yes	0.513	0.342–0.766	0.001
Lack of concentration			
Little or not at all	1.000		
Not too much	0.674	0.387–1.153	0.156
Sometimes or half the time	0.461	0.250–0.822	0.010
Nost of the time	0.877	0.455–1.666	0.690
Feeling hopeful			
Little or not at all	1.000		
Not too much	0.632	0.318–1.224	0.181
Sometimes or half the time	1.143	0.597–2.169	0.685
Most of the time	0.747	0.476–1.172	0.205
Self-rated health status			
Bad	1.000		
Fair	0.600	0.381–0.943	0.027
Good	0.363	0.196–0.658	0.001
Education level			
Elementary school and below	1.000		
Middle school	0.584	0.287–1.131	0.123
High school	0.507	0.178–1.246	0.164
College and above	2.027	0.069–24.746	0.629
Residence			
Rural	1.000		
Urban–rural	0.265	0.059–0.853	0.045
Urban	0.222	0.099–0.455	<0.001
Social interaction			
No	1.000		
Yes	0.669	0.446–0.998	0.050
Moderate physical activity			
No	1.000		
Yes	0.811	0.411–1.573	0.540
Mild physical activity			
No	1.000		
Yes	1.481	0.915–2.437	0.115
Health insurance			
No	1.000		
Yes	0.203	0.103–0.370	0.000

OR, odds ratio; CI, confidence interval.

### 3.3 Development of a dementia prediction model

**Figure 2:**
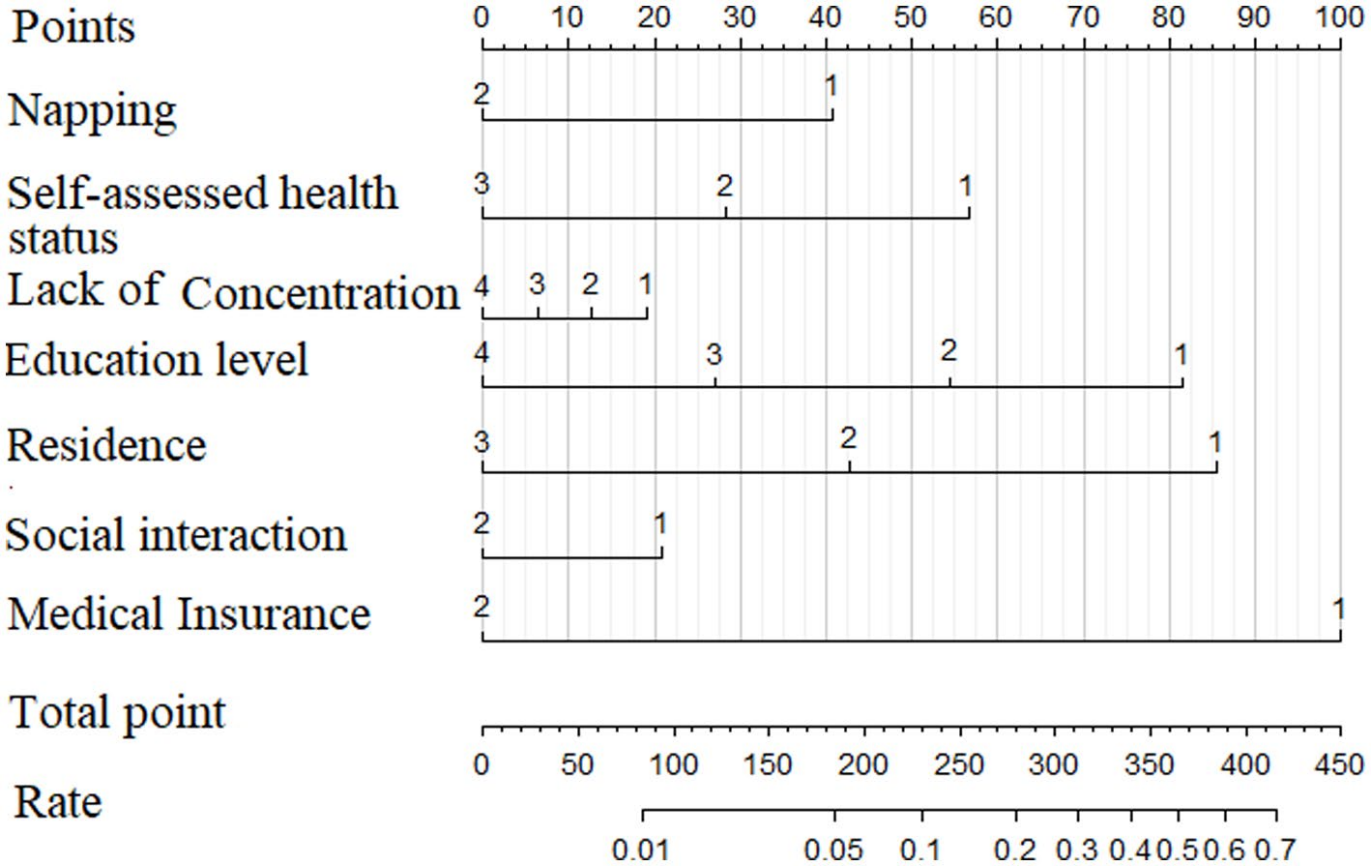
Dementia prediction model.

A dementia prediction model was developed through multifactorial binary logistic regression analysis, incorporating indicators including napping, lack of concentration, self-assessed health status, education level, residence, social interaction and medical insurance. As illustrated in Figure 2, the individual score of each patient can be calculated based on their specific conditions, using the corresponding scores for each indicator from the top axis. Once the total score is obtained by summing these individual scores, the risk probability of developing dementia can then be derived from the corresponding rate in the bottom axis.

### 3.4 Validation of the dementia prediction model

**Figure 3:**
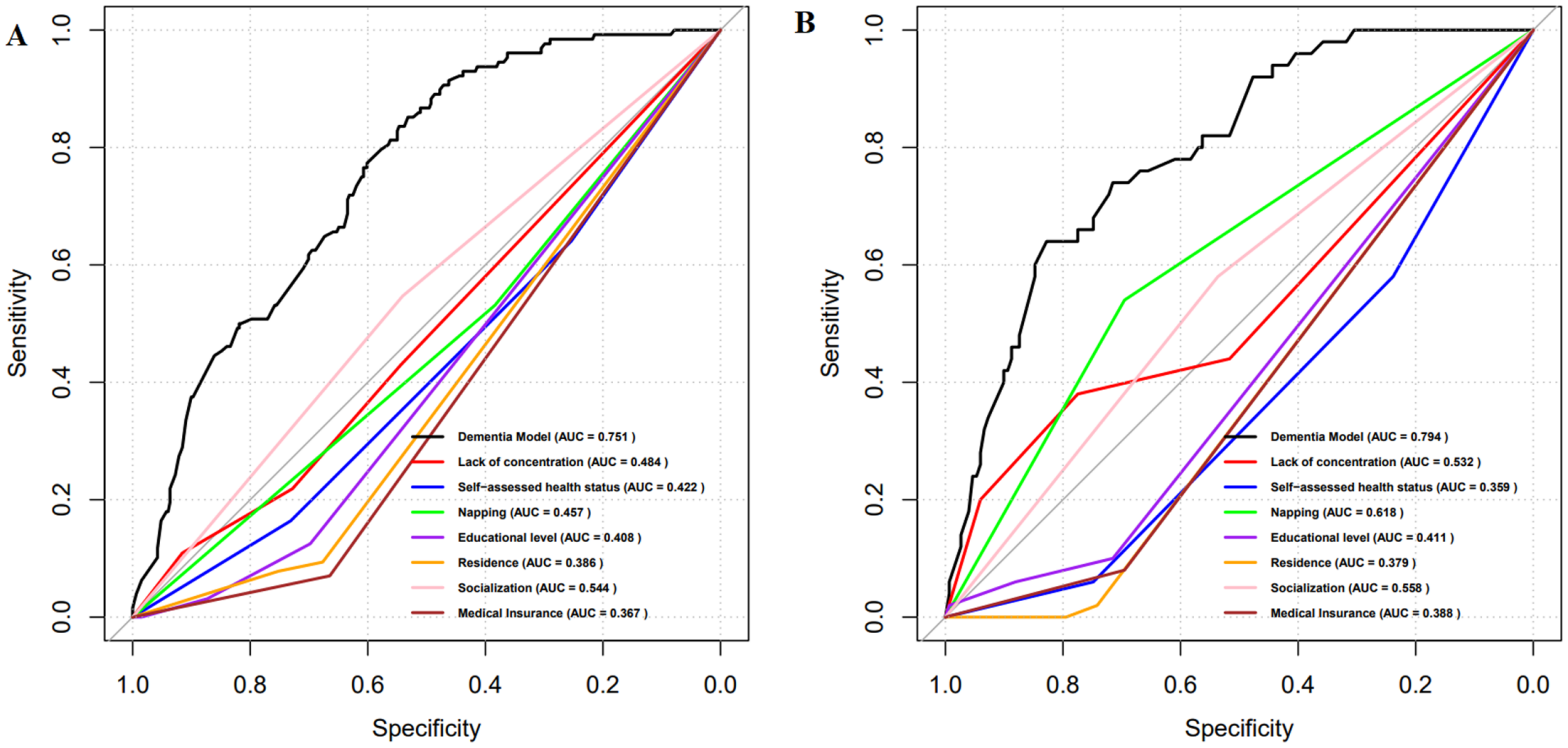
ROC curves for the dementia prediction model. **(A)** training set; **(B)** test set. ROC, Receptor operating characteristic curve; AUC, Area under the curve.

**Figure 4:**
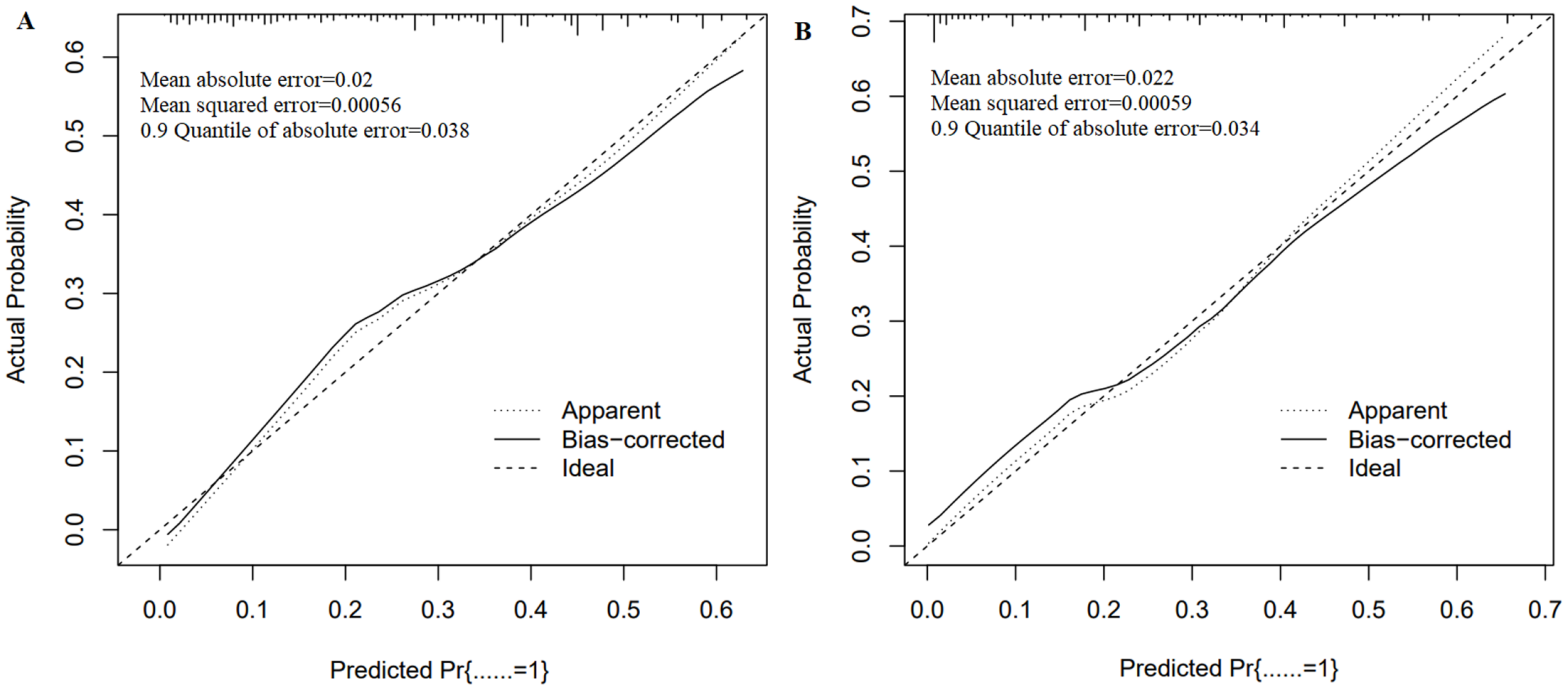
Calibration curves of the dementia prediction model. **(A)** training set; **(B)** test set.

**Figure 5:**
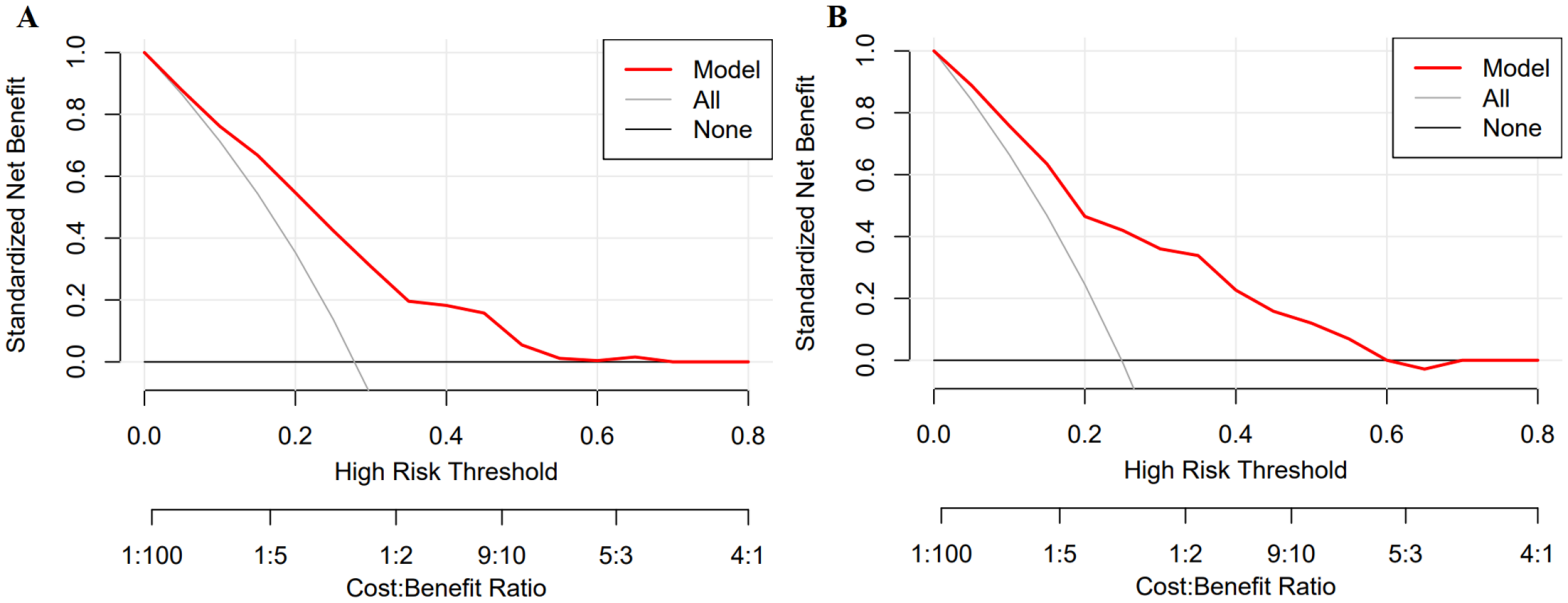
Clinical decision curve of the dementia prediction model. **(A)** training set; **(B)** test set.

The ROC analysis results presented in Figure 3 indicate that the AUC for the training set is 0.751, while the AUC for the test set is 0.794. The ROCs of both the training and test sets exhibit a greater discriminative capacity, suggesting that the proposed model is effective in distinguishing individuals with dementia from those without. The results of the DCA, as illustrated in Figure 4, reveal that the threshold probabilities for the training set range from 5 to 60%, while the threshold probabilities for the test set range from 1 to 60%. These findings imply that the proposed model demonstrates significant utility in risk identification and prediction. Calibration curves based on the logistic model showcased a high level of concordance with the predicted curves for both the training and testing sets, with minimal deviation from the ideal curve. These findings indicate the accuracy of the dementia prediction model in assessing the risk of developing dementia in the older adult (Figure 5).

## 4 Discussion

Focusing on a collective perspective, this study employed statistical methodologies like chi-square tests, LASSO regression analysis, and binary logistic regression analysis for a thorough exploration of key risk factors and underlying mechanisms contributing to dementia among the older adult population in Weifang. Subsequently, based on the findings, a nomogram prediction model for dementia, which encompasses biological, psychological, and social factors was developed utilizing the biopsychosocial model. As an empirical investigation focused on the older adult demographic, the goal of the study was to construct a dementia risk prediction model from a collective standpoint.

The outcomes of the LASSO and binary logistic regression analyses uncovered that napping, lack of concentration, self-assessed health status, education level, residence, social interaction and medical insurance were identified as significant factors influencing dementia. These findings align with prior research (9, 10, 12, 18, 20). The biopsychosocial model asserts that the onset of diseases, particularly mental illnesses like dementia, is influenced by a multitude of factors, including not only individual characteristics and psychological conditions but also the social environment. Considering the intricate nature of the onset mechanism for dementia, it is imperative to conduct a comprehensive analysis of the diverse risk factors influencing the occurrence of the disease. This is essential for enhancing the early detection rate of dementia and implementing proactive interventions for high-risk individuals. This enables healthcare professionals to assess dementia risk more accurately in the older adult, facilitating the prompt implementation of appropriate intervention measures.

The results of the study suggest that napping, as a biological factor within the biopsychosocial model, may be associated with a reduced risk of dementia. In China, where napping has a long-standing cultural tradition, the habit of taking short naps, often around 30 min per day, is established from childhood and tends to persist into old age. This practice is also prevalent in Weifang City and reflects broader societal norms regarding daily rest. Moderate napping has been shown to have significant benefits for the physical and mental well-being of the middle-aged and older adult populations (38). Specifically, moderate napping contributes to the consolidation of short-term memory and supports the brain in processing and storing new information. This is particularly relevant for older adults, whose cognitive functions tend to decline with age (39). Additionally, since the quality of nighttime sleep is often poorer in older individuals, napping can help to compensate for sleep deficits, alleviate daytime fatigue, and enhance attention and concentration (40, 41). Therefore, appropriate napping is recommended for older adults as a preventive measure against cognitive decline and dementia. Results of the study indicate that, as psychological factors within the biopsychosocial model, occasional inattentiveness and good self-rated health status are associated with dementia. In Weifang, many older adult individuals do not completely withdraw from social life after retirement but continue to engage in family affairs, such as caring for their grandchildren (42). While taking care of their grandchildren, older adults must allocate their attention and energy, managing household responsibilities, which may occasionally lead to lapses in attention. Moderate cognitive load can have a protective effect on the brain. Regularly adjusting and organizing daily tasks, as well as participating in various activities, helps enhance cognitive reserve and resilience (43). Self-rated health status is a culturally and biologically relevant indicator that reflects an individual’s overall physical and mental well-being (44). Studies have shown that individuals with poor self-rated health are more likely to develop dementia, whereas those with good self-rated health have a lower risk (45). This is likely because poor self-rated health is often associated with chronic conditions, which have been identified as significant risk factors for dementia (46). Therefore, promoting physical health among the older adult may contribute to reducing dementia incidence. Social factors such as educational level, place of residence, social interaction and medical insurance coverage are also closely linked to dementia risk. Lower educational attainment has been recognized as a risk factor for dementia, consistent with previous findings (10). Higher levels of education are associated with greater cognitive engagement in daily life and work, which provides continuous intellectual stimulation that helps maintain brain health and delay cognitive decline, ultimately reducing dementia risk (47). Our findings reveal that the prevalence of dementia is lower in urban areas compared to rural regions. This disparity may be attributed to differences in disease burden and access to affordable, timely, and acceptable healthcare services (48). Rural areas, with their limited healthcare resources, tend to exhibit a higher incidence of dementia. Additionally, older adults in rural areas often have lower educational levels and weaker health awareness. They may delay seeking medical attention and instead adopt a “wait-it-out” approach, leading to poor management of chronic conditions, which over time may negatively impact brain health and increase dementia risk (49). Participating in social activities such as square dancing and playing mahjong can provide emotional support for the older adult, reduce loneliness and depressive symptoms, stimulate cognitive function, and slow cognitive decline (50). In this regard, the Weifang government should increase investment in senior activity centers and universities for the older adult to enhance cognitive health among the older adult population. The study also identified an association between the absence of health insurance and an increased risk of dementia, which is consistent with previous studies (20). In response to China’s “Healthy China 2030” strategy, the government of Weifang has introduced policies to improve medical insurance benefits and provide allowances for the older adult. However, some older adult individuals remain reluctant to enroll in medical insurance programs. The lack of health insurance may result in delays or avoidance of seeking medical care, potentially leading to the neglect of early symptoms such as mild cognitive impairment and missed opportunities for early intervention. Therefore, efforts should continue to expand health insurance coverage and promote more equitable access to healthcare services to prevent the onset and progression of dementia.

Grounded in the biopsychosocial model, a dementia prediction model was developed, exhibiting excellent discriminative, calibration, and predictive validity. These findings indicate that the model is valuable for the collective screening of dementia risk in the older adult population. By providing the probability of dementia development for different populations based on various variables, the model is capable of quantifying dementia risk and effectively predicting the onset of the disease. In addition, the proposed model boasts several advantages over those listed in prior research. First, the data required by the model is easily accessible. Integrating psychological and social factors, the model relies on data that is simpler to obtain compared to traditional clinical biological data. Clinical data often necessitates complex medical tests and specialized equipment, whereas psychological and social data can be acquired through straightforward questionnaires, interviews, or existing social research materials (51). Second, the model incurs low application costs. As it does not demand expensive medical tests and specialized equipment, the implementation cost of this model is significantly lower than those relying on clinical biological data (52). Third, this model is conducive to home-based self-assessment by older adult individuals. Primarily relying on psychological and social factors, the proposed model allows older adult individuals to self-assess dementia risk through uncomplicated questionnaires or online tools in their home environment (53). Lastly, the model is suitable for collective dementia screening (11). Unlike models relying on clinical biological data, this model solely requires simple questionnaires or interviews, eliminating the need for costly medical equipment. This renders the model more applicable in large-scale dementia screening for the older adult population.

Nevertheless, certain limitations should be acknowledged. First, the sample size of this study is relatively small, which may affect the robustness and generalizability of the findings. Additionally, as the data were derived from the Weifang population in China, its applicability to older adult populations in other countries and regions could be constrained. Future endeavors will focus on broadening both the sample size and geographical scope to provide a more universally effective reference tool for dementia screening in diverse older adult populations.

## 5 Conclusion

A nomogram prediction model for dementia risk was established utilizing the biopsychosocial model. Regarding data accessibility, cost-effectiveness, availability of self-assessment for seniors at home, and suitability for group risk screening, the proposed model exhibits substantial advantages over traditional prediction models primarily reliant on biological data. It stands out as a more practical and efficient tool for the early identification and intervention of dementia in the older adult population.

## Data Availability

The raw data supporting the conclusions of this article will be made available by the authors, without undue reservation.
