# Supplementary material for: Development of a nomogram model for predicting dementia risk in the older adult population of Weifang, Shandong Province, China: based on the biopsychosocial model
Source: Front Public Health. 2025 Feb 21;13:1499820. doi: 10.3389/fpubh.2025.1499820 (PMC11885151; doi:10.3389/fpubh.2025.1499820)
Supplement: Supplementary file 1 [file Data_Sheet_1.docx]

# APPENDIX

**Supplementary Table 1.** Details of variable assignment.

| Dimension | Variable | Assignment |
| --- | --- | --- |
| Biological Factors | Gender | 1=Male; 2=Female |
|  | Age | 1=60~69 years; 2=70~79 years; 3=80~89 years; 4=≥90 years |
|  | Physical Disability | 1=No; 2=Yes |
|  | Brain Injury | 1=No; 2=Yes |
|  | Visual Impairment | 1=No; 2=Yes |
|  | Hearing Impairment | 1=No; 2=Yes |
|  | Language Dysfunctio | 1=No; 2=Yes |
|  | Hypertension | 1=No; 2=Yes |
|  | Hyperlipidemia | 1=No; 2=Yes |
|  | Diabetes | 1=No; 2=Yes |
|  | Cancer | 1=No; 2=Yes |
|  | Chronic Pulmonary Disease | 1=No; 2=Yes |
|  | Liver Disease | 1=No; 2=Yes |
|  | Heart Disease | 1=No; 2=Yes |
|  | Stroke | 1=No; 2=Yes |
|  | Kidney Disease | 1=No; 2=Yes |
|  | Arthritis | 1=No; 2=Yes |
|  | Asthma | 1=No; 2=Yes |
|  | Sleep Duration | 1=≤5 hours; 2=6 to 8 hours, 3=≥9 hours |
|  | Napping | 1=No; 2=Yes |
| Psychological Factors | Annoyed by minor things | 1 = little or not at all; 2 = not too much; 3 = sometimes or half the time; 4 = most of the time |
|  | Lack of Concentration | 1 = little or not at all; 2 = not too much; 3 = sometimes or half the time; 4 = most of the time |
|  | Feeling Down | 1 = little or not at all; 2 = not too much; 3 = sometimes or half the time; 4 = most of the time |
|  | Difficulty Doing Things | 1 = little or not at all; 2 = not too much; 3 = sometimes or half the time; 4 = most of the time |
|  | Feeling Hopeful | 1 = little or not at all; 2 = not too much; 3 = sometimes or half the time; 4 = most of the time |
|  | Feeling Afraid | 1 = little or not at all; 2 = not too much; 3 = sometimes or half the time; 4 = most of the time |
|  | Poor Sleep Quality | 1 = little or not at all; 2 = not too much; 3 = sometimes or half the time; 4 = most of the time |
|  | Feeling Happy | 1 = little or not at all; 2 = not too much; 3 = sometimes or half the time; 4 = most of the time |
|  | Feeling Lonely | 1 = little or not at all; 2 = not too much; 3 = sometimes or half the time; 4 = most of the time |
|  | Negative Thinking | 1 = little or not at all; 2 = not too much; 3 = sometimes or half the time; 4 = most of the time |
|  | Self-rated Health Status | 1=bad; 2=fair; 3=good |
|  | Emotional and Mental Issues | 1=No; 2=Yes |
|  | Smoking | 1=No; 2=Yes |
|  | Drinking Alcohol | 1=No; 2=Yes |
| Social Factors | Education level | 1=Elementary school and below; 2=Middle school; 3=High school; 4=College and above |
|  | Marital Status | 1=Married; 2=Unmarried |
|  | Residence | 1=rural; 2=urban-rural; 3=urban |
|  | Living Alone | 1=No; 2=Yes |
|  | Social interaction | 1=No; 2=Yes |
|  | Intense Physical Activity | 1=No; 2=Yes |
|  | Moderate Physical Activity | 1=No; 2=Yes |
|  | Light Physical Activity | 1=No; 2=Yes |
|  | Physical Examination | 1=No; 2=Yes |
|  | Medical Insurance | 1=No; 2=Yes |
|  | Community Elderly Care Services | 1=No; 2=Yes |

**Supplementary Table 2.** Alzheimer's baseline analysis.

| Variable | Total (n = 660) | Alzheimer | | *χ²* | *p* |
| --- | --- | --- | --- | --- | --- |
|  |  | 0 (n = 482) | 1 (n = 178) |  |  |
| Gender |  |  |  | 2.582 | 0.108 |
| 1 | 338 (51.21) | 256 (53.11) | 82 (46.07) |  |  |
| 2 | 322 (48.79) | 226 (46.89) | 96 (53.93) |  |  |
| Age |  |  |  | 0.389 | 0.943 |
| 1 | 406 (61.52) | 298 (61.83) | 108 (60.67) |  |  |
| 2 | 198 (30) | 145 (30.08) | 53 (29.78) |  |  |
| 3 | 50 (7.58) | 35 (7.26) | 15 (8.43) |  |  |
| 4 | 6 (0.91) | 4 (0.83) | 2 (1.12) |  |  |
| Physical Disability |  |  |  | 1.772 | 0.183 |
| 1 | 639 (96.82) | 464 (96.27) | 175 (98.31) |  |  |
| 2 | 21 (3.18) | 18 (3.73) | 3 (1.69) |  |  |
| Brain Injury |  |  |  | 0.641 | 0.423 |
| 1 | 635 (96.21) | 462 (95.85) | 173 (97.19) |  |  |
| 2 | 25 (3.79) | 20 (4.15) | 5 (2.81) |  |  |
| Visual Impairment |  |  |  | 1.850 | 0.174 |
| 1 | 631 (95.61) | 464 (96.27) | 167 (93.82) |  |  |
| 2 | 29 (4.39) | 18 (3.73) | 11 (6.18) |  |  |
| Hearing Impairment |  |  |  | 1.677 | 0.195 |
| 1 | 621 (94.09) | 457 (94.81) | 164 (92.13) |  |  |
| 2 | 39 (5.91) | 25 (5.19) | 14 (7.87) |  |  |
| Language Dysfunction |  |  |  | 0.492 | 0.483 |
| 1 | 651 (98.64) | 474 (98.34) | 177 (99.44) |  |  |
| 2 | 9 (1.36) | 8 (1.66) | 1 (0.56) |  |  |
| Hypertension |  |  |  | 0.087 | 0.768 |
| 1 | 557 (84.39) | 408 (84.65) | 149 (83.71) |  |  |
| 2 | 103 (15.61) | 74 (15.35) | 29 (16.29) |  |  |
| Hyperlipidemia |  |  |  | 2.439 | 0.118 |
| 1 | 609 (92.27) | 440 (91.29) | 169 (94.94) |  |  |
| 2 | 51 (7.73) | 42 (8.71) | 9 (5.06) |  |  |
| Diabetes |  |  |  | 1.191 | 0.275 |
| 1 | 615 (93.18) | 446 (92.53) | 169 (94.94) |  |  |
| 2 | 45 (6.82) | 36 (7.47) | 9 (5.06) |  |  |
| Cancer |  |  |  | 1.765 | 0.184 |
| 1 | 652 (98.79) | 474 (98.34) | 178 (100.00) |  |  |
| 2 | 8 (1.21) | 8 (1.66) | 0 (0.00) |  |  |
| Chronic Pulmonary Disease |  |  |  | 2.052 | 0.152 |
| 1 | 619 (93.79) | 456 (94.61) | 163 (91.57) |  |  |
| 2 | 41 (6.21) | 26 (5.39) | 15 (8.43) |  |  |
| Liver Disease |  |  |  | 0.049 | 0.825 |
| 1 | 636 (96.36) | 464 (96.27) | 172 (96.63) |  |  |
| 2 | 24 (3.64) | 18 (3.73) | 6 (3.37) |  |  |
| Heart Disease |  |  |  | 1.776 | 0.183 |
| 1 | 612 (92.73) | 443 (91.91) | 169 (94.94) |  |  |
| 2 | 48 (7.27) | 39 (8.09) | 9 (5.06) |  |  |
| Stroke |  |  |  | 1.154 | 0.283 |
| 1 | 628 (95.15) | 456 (94.61) | 172 (96.63) |  |  |
| 2 | 32 (4.85) | 26 (5.39) | 6 (3.37) |  |  |
| Kidney Disease |  |  |  | 0.254 | 0.614 |
| 1 | 631 (95.61) | 462 (95.85) | 169 (94.94) |  |  |
| 2 | 29 (4.39) | 20 (4.15) | 9 (5.06) |  |  |
| Arthritis |  |  |  | 4.550 | 0.033 |
| 1 | 588 (89.09) | 437 (90.66) | 151 (84.83) |  |  |
| 2 | 72 (10.91) | 45 (9.34) | 27 (15.17) |  |  |
| Asthma |  |  |  | 0.000 | 1.000 |
| 1 | 653 (98.94) | 477 (98.96) | 176 (98.88) |  |  |
| 2 | 7 (1.06) | 5 (1.04) | 2 (1.12) |  |  |
| Sleep Duration |  |  |  | 6.859 | 0.032 |
| 1 | 198 (30) | 131 (27.18) | 67 (37.64) |  |  |
| 2 | 387 (58.64) | 293 (60.79) | 94 (52.81) |  |  |
| 3 | 75 (11.36) | 58 (12.03) | 17 (9.55) |  |  |
| Napping |  |  |  | 9.179 | 0.002 |
| 1 | 260 (39.39) | 173 (35.89) | 87 (48.88) |  |  |
| 2 | 400 (60.61) | 309 (64.11) | 91 (51.12) |  |  |
| Annoyed by minor things |  |  |  | 6.372 | 0.095 |
| 1 | 363 (55) | 268 (55.60) | 95 (53.37) |  |  |
| 2 | 120 (18.18) | 94 (19.50) | 26 (14.61) |  |  |
| 3 | 97 (14.7) | 70 (14.52) | 27 (15.17) |  |  |
| 4 | 80 (12.12) | 50 (10.37) | 30 (16.85) |  |  |
| Lack of Concentration |  |  |  | 8.041 | 0.045 |
| 1 | 359 (54.39) | 258 (53.53) | 101 (56.74) |  |  |
| 2 | 130 (19.7) | 100 (20.75) | 30 (16.85) |  |  |
| 3 | 110 (16.67) | 87 (18.05) | 23 (12.92) |  |  |
| 4 | 61 (9.24) | 37 (7.68) | 24 (13.48) |  |  |
| Feeling Down |  |  |  | 5.113 | 0.164 |
| 1 | 362 (54.85) | 263 (54.56) | 99 (55.62) |  |  |
| 2 | 112 (16.97) | 90 (18.67) | 22 (12.36) |  |  |
| 3 | 109 (16.52) | 73 (15.15) | 36 (20.22) |  |  |
| 4 | 77 (11.67) | 56 (11.62) | 21 (11.80) |  |  |
| Difficulty Doing Things |  |  |  | 0.806 | 0.848 |
| 1 | 345 (52.27) | 253 (52.49) | 92 (51.69) |  |  |
| 2 | 111 (16.82) | 84 (17.43) | 27 (15.17) |  |  |
| 3 | 93 (14.09) | 66 (13.69) | 27 (15.17) |  |  |
| 4 | 111 (16.82) | 79 (16.39) | 32 (17.98) |  |  |
| Feeling Hopeful |  |  |  | 5.886 | 0.117 |
| 1 | 219 (33.18) | 150 (31.12) | 69 (38.76) |  |  |
| 2 | 81 (12.27) | 63 (13.07) | 18 (10.11) |  |  |
| 3 | 86 (13.03) | 59 (12.24) | 27 (15.17) |  |  |
| 4 | 274 (41.52) | 210 (43.57) | 64 (35.96) |  |  |
| Feeling Afraid |  |  |  | 0.872 | 0.832 |
| 1 | 517 (78.33) | 378 (78.42) | 139 (78.09) |  |  |
| 2 | 60 (9.09) | 46 (9.54) | 14 (7.87) |  |  |
| 3 | 45 (6.82) | 31 (6.43) | 14 (7.87) |  |  |
| 4 | 38 (5.76) | 27 (5.60) | 11 (6.18) |  |  |
| Poor Sleep Quality |  |  |  | 8.351 | 0.039 |
| 1 | 324 (49.09) | 248 (51.45) | 76 (42.70) |  |  |
| 2 | 104 (15.76) | 71 (14.73) | 33 (18.54) |  |  |
| 3 | 97 (14.7) | 75 (15.56) | 22 (12.36) |  |  |
| 4 | 135 (20.45) | 88 (18.26) | 47 (26.40) |  |  |
| Feeling Happy |  |  |  | 2.957 | 0.398 |
| 1 | 131 (19.85) | 89 (18.46) | 42 (23.60) |  |  |
| 2 | 86 (13.03) | 65 (13.49) | 21 (11.80) |  |  |
| 3 | 99 (15) | 70 (14.52) | 29 (16.29) |  |  |
| 4 | 344 (52.12) | 258 (53.53) | 86 (48.31) |  |  |
| Feeling Lonely |  |  |  | 4.690 | 0.196 |
| 1 | 455 (68.94) | 336 (69.71) | 119 (66.85) |  |  |
| 2 | 76 (11.52) | 59 (12.24) | 17 (9.55) |  |  |
| 3 | 56 (8.48) | 41 (8.51) | 15 (8.43) |  |  |
| 4 | 73 (11.06) | 46 (9.54) | 27 (15.17) |  |  |
| Negative Thinking |  |  |  | 3.202 | 0.361 |
| 1 | 516 (78.18) | 385 (79.88) | 131 (73.60) |  |  |
| 2 | 55 (8.33) | 38 (7.88) | 17 (9.55) |  |  |
| 3 | 40 (6.06) | 26 (5.39) | 14 (7.87) |  |  |
| 4 | 49 (7.42) | 33 (6.85) | 16 (8.99) |  |  |
| Self-rated Health Status |  |  |  | 16.857 | <0.001 |
| 1 | 187 (28.33) | 120 (24.90) | 67 (37.64) |  |  |
| 2 | 322 (48.79) | 235 (48.76) | 87 (48.88) |  |  |
| 3 | 151 (22.88) | 127 (26.35) | 24 (13.48) |  |  |
| Emotional and Mental Issues |  |  |  | 0.000 | 1.000 |
| 1 | 655 (99.24) | 478 (99.17) | 177 (99.44) |  |  |
| 2 | 5 (0.76) | 4 (0.83) | 1 (0.56) |  |  |
| Smoking |  |  |  | 0.079 | 0.778 |
| 1 | 582 (88.18) | 424 (87.97) | 158 (88.76) |  |  |
| 2 | 78 (11.82) | 58 (12.03) | 20 (11.24) |  |  |
| Drinking Alcohol |  |  |  | 1.541 | 0.215 |
| 1 | 454 (68.79) | 325 (67.43) | 129 (72.47) |  |  |
| 2 | 206 (31.21) | 157 (32.57) | 49 (27.53) |  |  |
| Educational Level |  |  |  | 22.608 | <0.001 |
| 1 | 496 (75.15) | 339 (70.33) | 157 (88.20) |  |  |
| 2 | 97 (14.7) | 83 (17.22) | 14 (7.87) |  |  |
| 3 | 60 (9.09) | 54 (11.20) | 6 (3.37) |  |  |
| 4 | 7 (1.06) | 6 (1.24) | 1 (0.56) |  |  |
| Marital Status |  |  |  | 3.832 | 0.050 |
| 1 | 137 (20.76) | 91 (18.88) | 46 (25.84) |  |  |
| 2 | 523 (79.24) | 391 (81.12) | 132 (74.16) |  |  |
| Residence |  |  |  | 37.562 | <0.001 |
| 1 | 501 (75.91) | 336 (69.71) | 165 (92.70) |  |  |
| 2 | 36 (5.45) | 33 (6.85) | 3 (1.69) |  |  |
| 3 | 123 (18.64) | 113 (23.44) | 10 (5.62) |  |  |
| Living Alone |  |  |  | 0.002 | 0.969 |
| 1 | 511 (77.42) | 373 (77.39) | 138 (77.53) |  |  |
| 2 | 149 (22.58) | 109 (22.61) | 40 (22.47) |  |  |
| Socializing |  |  |  | 4.756 | 0.029 |
| 1 | 321 (48.64) | 222 (46.06) | 99 (55.62) |  |  |
| 2 | 339 (51.36) | 260 (53.94) | 79 (44.38) |  |  |
| Intense Physical Activity |  |  |  | 2.426 | 0.119 |
| 1 | 471 (71.36) | 352 (73.03) | 119 (66.85) |  |  |
| 2 | 189 (28.64) | 130 (26.97) | 59 (33.15) |  |  |
| Moderate Physical Activity |  |  |  | 2.453 | 0.293 |
| 0 | 182 (27.58) | 125 (25.93) | 57 (32.02) |  |  |
| 1 | 394 (59.7) | 295 (61.20) | 99 (55.62) |  |  |
| 2 | 84 (12.73) | 62 (12.86) | 22 (12.36) |  |  |
| Light Physical Activity |  |  |  | 0.199 | 0.656 |
| 1 | 145 (21.97) | 108 (22.41) | 37 (20.79) |  |  |
| 2 | 515 (78.03) | 374 (77.59) | 141 (79.21) |  |  |
| Physical Examination |  |  |  | 5.107 | 0.024 |
| 1 | 26 (3.94) | 24 (4.98) | 2 (1.12) |  |  |
| 2 | 634 (96.06) | 458 (95.02) | 176 (98.88) |  |  |
| Medical Insurance |  |  |  | 43.406 | <0.001 |
| 1 | 490 (74.24) | 325 (67.43) | 165 (92.70) |  |  |
| 2 | 170 (25.76) | 157 (32.57) | 13 (7.30) |  |  |
| Community Elderly Care Services |  |  |  | 0.326 | 0.568 |
| 1 | 490 (74.24) | 355 (73.65) | 135 (75.84) |  |  |
| 2 | 170 (25.76) | 127 (26.35) | 43 (24.16) |  |  |

**Supplementary Table 3.** Homogeneity test for Alzheimer's training and test sets.

| Variable | Total (n = 660) | Training Set (n = 459) | Test Set (n = 201) | *χ²* | *P* |
| --- | --- | --- | --- | --- | --- |
| Alzheimer |  |  |  | 0.643 | 0.422 |
| 0 | 482 (73.03) | 331 (72.11) | 151 (75.12) |  |  |
| 1 | 178 (26.97) | 128 (27.89) | 50 (24.88) |  |  |
| Gender |  |  |  | 0.269 | 0.604 |
| 1 | 338 (51.21) | 232 (50.54) | 106 (52.74) |  |  |
| 2 | 322 (48.79) | 227 (49.46) | 95 (47.26) |  |  |
| Age |  |  |  | 0.269 | 0.831 |
| 1 | 406 (61.52) | 279 (60.78) | 127 (63.18) |  |  |
| 2 | 198 (30) | 138 (30.07) | 60 (29.85) |  |  |
| 3 | 50 (7.58) | 37 (8.06) | 13 (6.47) |  |  |
| 4 | 6 (0.91) | 5 (1.09) | 1 (0.50) |  |  |
| Physical Disability |  |  |  | 0.036 | 0.849 |
| 1 | 639 (96.82) | 444 (96.73) | 195 (97.01) |  |  |
| 2 | 21 (3.18) | 15 (3.27) | 6 (2.99) |  |  |
| Brain Injury |  |  |  | 0.511 | 0.475 |
| 1 | 635 (96.21) | 440 (95.86) | 195 (97.01) |  |  |
| 2 | 25 (3.79) | 19 (4.14) | 6 (2.99) |  |  |
| Visual Impairment |  |  |  | 0.801 | 0.371 |
| 1 | 631 (95.61) | 441 (96.08) | 190 (94.53) |  |  |
| 2 | 29 (4.39) | 18 (3.92) | 11 (5.47) |  |  |
| Hearing Impairment |  |  |  | 1.255 | 0.263 |
| 1 | 621 (94.09) | 435 (94.77) | 186 (92.54) |  |  |
| 2 | 39 (5.91) | 24 (5.23) | 15 (7.46) |  |  |
| Language Dysfunction |  |  |  | 0.031 | 0.861 |
| 1 | 651 (98.64) | 452 (98.47) | 199 (99.00) |  |  |
| 2 | 9 (1.36) | 7 (1.53) | 2 (1.00) |  |  |
| Hypertension |  |  |  | 1.165 | 0.280 |
| 1 | 557 (84.39) | 392 (85.40) | 165 (82.09) |  |  |
| 2 | 103 (15.61) | 67 (14.60) | 36 (17.91) |  |  |
| Hyperlipidemia |  |  |  | 0.611 | 0.434 |
| 1 | 609 (92.27) | 426 (92.81) | 183 (91.04) |  |  |
| 2 | 51 (7.73) | 33 (7.19) | 18 (8.96) |  |  |
| Diabetes |  |  |  | 0.056 | 0.813 |
| 1 | 615 (93.18) | 427 (93.03) | 188 (93.53) |  |  |
| 2 | 45 (6.82) | 32 (6.97) | 13 (6.47) |  |  |
| Cancer |  |  |  | 0.000 | 1.000 |
| 1 | 652 (98.79) | 453 (98.69) | 199 (99.00) |  |  |
| 2 | 8 (1.21) | 6 (1.31) | 2 (1.00) |  |  |
| Chronic Pulmonary Disease |  |  |  | 0.271 | 0.602 |
| 1 | 619 (93.79) | 429 (93.46) | 190 (94.53) |  |  |
| 2 | 41 (6.21) | 30 (6.54) | 11 (5.47) |  |  |
| Liver Disease |  |  |  | 0.584 | 0.445 |
| 1 | 636 (96.36) | 444 (96.73) | 192 (95.52) |  |  |
| 2 | 24 (3.64) | 15 (3.27) | 9 (4.48) |  |  |
| Heart Disease |  |  |  | 4.320 | 0.038 |
| 1 | 612 (92.73) | 432 (94.12) | 180 (89.55) |  |  |
| 2 | 48 (7.27) | 27 (5.88) | 21 (10.45) |  |  |
| Stroke |  |  |  | 0.010 | 0.920 |
| 1 | 628 (95.15) | 437 (95.21) | 191 (95.02) |  |  |
| 2 | 32 (4.85) | 22 (4.79) | 10 (4.98) |  |  |
| Kidney Disease |  |  |  | 1.709 | 0.191 |
| 1 | 631 (95.61) | 442 (96.30) | 189 (94.03) |  |  |
| 2 | 29 (4.39) | 17 (3.70) | 12 (5.97) |  |  |
| Arthritis |  |  |  | 0.695 | 0.404 |
| 1 | 588 (89.09) | 412 (89.76) | 176 (87.56) |  |  |
| 2 | 72 (10.91) | 47 (10.24) | 25 (12.44) |  |  |
| Asthma |  |  |  | 0.272 | 0.602 |
| 1 | 653 (98.94) | 453 (98.69) | 200 (99.50) |  |  |
| 2 | 7 (1.06) | 6 (1.31) | 1 (0.50) |  |  |
| Sleep Duration |  |  |  | 1.534 | 0.464 |
| 1 | 198 (30) | 131 (28.54) | 67 (33.33) |  |  |
| 2 | 387 (58.64) | 275 (59.91) | 112 (55.72) |  |  |
| 3 | 75 (11.36) | 53 (11.55) | 22 (10.95) |  |  |
| Napping |  |  |  | 1.145 | 0.285 |
| 1 | 260 (39.39) | 187 (40.74) | 73 (36.32) |  |  |
| 2 | 400 (60.61) | 272 (59.26) | 128 (63.68) |  |  |
| Annoyed by minor things |  |  |  | 7.515 | 0.057 |
| 1 | 363 (55) | 238 (51.85) | 125 (62.19) |  |  |
| 2 | 120 (18.18) | 94 (20.48) | 26 (12.94) |  |  |
| 3 | 97 (14.7) | 70 (15.25) | 27 (13.43) |  |  |
| 4 | 80 (12.12) | 57 (12.42) | 23 (11.44) |  |  |
| Lack of Concentration |  |  |  | 0.381 | 0.944 |
| 1 | 359 (54.39) | 253 (55.12) | 106 (52.74) |  |  |
| 2 | 130 (19.7) | 88 (19.17) | 42 (20.90) |  |  |
| 3 | 110 (16.67) | 76 (16.56) | 34 (16.92) |  |  |
| 4 | 61 (9.24) | 42 (9.15) | 19 (9.45) |  |  |
| Feeling Down |  |  |  | 3.151 | 0.369 |
| 1 | 362 (54.85) | 247 (53.81) | 115 (57.21) |  |  |
| 2 | 112 (16.97) | 81 (17.65) | 31 (15.42) |  |  |
| 3 | 109 (16.52) | 72 (15.69) | 37 (18.41) |  |  |
| 4 | 77 (11.67) | 59 (12.85) | 18 (8.96) |  |  |
| Difficulty Doing Things |  |  |  | 1.380 | 0.710 |
| 1 | 345 (52.27) | 243 (52.94) | 102 (50.75) |  |  |
| 2 | 111 (16.82) | 77 (16.78) | 34 (16.92) |  |  |
| 3 | 93 (14.09) | 60 (13.07) | 33 (16.42) |  |  |
| 4 | 111 (16.82) | 79 (17.21) | 32 (15.92) |  |  |
| Feeling Hopeful |  |  |  | 9.679 | 0.021 |
| 1 | 219 (33.18) | 161 (35.08) | 58 (28.86) |  |  |
| 2 | 81 (12.27) | 45 (9.80) | 36 (17.91) |  |  |
| 3 | 86 (13.03) | 63 (13.73) | 23 (11.44) |  |  |
| 4 | 274 (41.52) | 190 (41.39) | 84 (41.79) |  |  |
| Feeling Afraid |  |  |  | 1.386 | 0.709 |
| 1 | 517 (78.33) | 355 (77.34) | 162 (80.60) |  |  |
| 2 | 60 (9.09) | 44 (9.59) | 16 (7.96) |  |  |
| 3 | 45 (6.82) | 34 (7.41) | 11 (5.47) |  |  |
| 4 | 38 (5.76) | 26 (5.66) | 12 (5.97) |  |  |
| Poor Sleep Quality |  |  |  | 6.193 | 0.103 |
| 1 | 324 (49.09) | 216 (47.06) | 108 (53.73) |  |  |
| 2 | 104 (15.76) | 68 (14.81) | 36 (17.91) |  |  |
| 3 | 97 (14.7) | 75 (16.34) | 22 (10.95) |  |  |
| 4 | 135 (20.45) | 100 (21.79) | 35 (17.41) |  |  |
| Feeling Happy |  |  |  | 1.477 | 0.688 |
| 1 | 131 (19.85) | 96 (20.92) | 35 (17.41) |  |  |
| 2 | 86 (13.03) | 61 (13.29) | 25 (12.44) |  |  |
| 3 | 99 (15) | 69 (15.03) | 30 (14.93) |  |  |
| 4 | 344 (52.12) | 233 (50.76) | 111 (55.22) |  |  |
| Feeling Lonely |  |  |  | 1.676 | 0.642 |
| 1 | 455 (68.94) | 314 (68.41) | 141 (70.15) |  |  |
| 2 | 76 (11.52) | 50 (10.89) | 26 (12.94) |  |  |
| 3 | 56 (8.48) | 42 (9.15) | 14 (6.97) |  |  |
| 4 | 73 (11.06) | 53 (11.55) | 20 (9.95) |  |  |
| Negative Thinking |  |  |  | 2.152 | 0.541 |
| 1 | 516 (78.18) | 355 (77.34) | 161 (80.10) |  |  |
| 2 | 55 (8.33) | 43 (9.37) | 12 (5.97) |  |  |
| 3 | 40 (6.06) | 27 (5.88) | 13 (6.47) |  |  |
| 4 | 49 (7.42) | 34 (7.41) | 15 (7.46) |  |  |
| Self-rated Health Status |  |  |  | 1.135 | 0.567 |
| 1 | 187 (28.33) | 130 (28.32) | 57 (28.36) |  |  |
| 2 | 322 (48.79) | 219 (47.71) | 103 (51.24) |  |  |
| 3 | 151 (22.88) | 110 (23.97) | 41 (20.40) |  |  |
| Emotional and Mental Issues |  |  |  | 0.000 | 1.000 |
| 1 | 655 (99.24) | 456 (99.35) | 199 (99.00) |  |  |
| 2 | 5 (0.76) | 3 (0.65) | 2 (1.00) |  |  |
| Smoking |  |  |  | 5.868 | 0.015 |
| 1 | 582 (88.18) | 414 (90.20) | 168 (83.58) |  |  |
| 2 | 78 (11.82) | 45 (9.80) | 33 (16.42) |  |  |
| Drinking Alcohol |  |  |  | 1.307 | 0.253 |
| 1 | 454 (68.79) | 322 (70.15) | 132 (65.67) |  |  |
| 2 | 206 (31.21) | 137 (29.85) | 69 (34.33) |  |  |
| Educational Level |  |  |  | 0.404 | 0.945 |
| 1 | 496 (75.15) | 343 (74.73) | 153 (76.12) |  |  |
| 2 | 97 (14.7) | 70 (15.25) | 27 (13.43) |  |  |
| 3 | 60 (9.09) | 41 (8.93) | 19 (9.45) |  |  |
| 4 | 7 (1.06) | 5 (1.09) | 2 (1.00) |  |  |
| Marital Status |  |  |  | 0.226 | 0.635 |
| 1 | 137 (20.76) | 93 (20.26) | 44 (21.89) |  |  |
| 2 | 523 (79.24) | 366 (79.74) | 157 (78.11) |  |  |
| Residence |  |  |  | 2.776 | 0.250 |
| 1 | 501 (75.91) | 340 (74.07) | 161 (80.10) |  |  |
| 2 | 36 (5.45) | 27 (5.88) | 9 (4.48) |  |  |
| 3 | 123 (18.64) | 92 (20.04) | 31 (15.42) |  |  |
| Living Alone |  |  |  | 1.294 | 0.255 |
| 1 | 511 (77.42) | 361 (78.65) | 150 (74.63) |  |  |
| 2 | 149 (22.58) | 98 (21.35) | 51 (25.37) |  |  |
| Socializing |  |  |  | 0.044 | 0.834 |
| 1 | 321 (48.64) | 222 (48.37) | 99 (49.25) |  |  |
| 2 | 339 (51.36) | 237 (51.63) | 102 (50.75) |  |  |
| Intense Physical Activity |  |  |  | 0.728 | 0.394 |
| 1 | 471 (71.36) | 323 (70.37) | 148 (73.63) |  |  |
| 2 | 189 (28.64) | 136 (29.63) | 53 (26.37) |  |  |
| Moderate Physical Activity |  |  |  | 2.979 | 0.084 |
| 0 | 266 (40.3) | 195 (42.48) | 71 (35.32) |  |  |
| 1 | 394 (59.7) | 264 (57.52) | 130 (64.68) |  |  |
| 2 |  |  |  | 0.722 | 0.396 |
| Light Physical Activity | 145 (21.97) | 105 (22.88) | 40 (19.90) |  |  |
| 1 | 515 (78.03) | 354 (77.12) | 161 (80.10) |  |  |
| 2 |  |  |  | 0.696 | 0.404 |
| Physical Examination | 26 (3.94) | 20 (4.36) | 6 (2.99) |  |  |
| 1 | 634 (96.06) | 439 (95.64) | 195 (97.01) |  |  |
| 2 |  |  |  | 0.118 | 0.732 |
| Medical Insurance | 490 (74.24) | 339 (73.86) | 151 (75.12) |  |  |
| 1 | 170 (25.76) | 120 (26.14) | 50 (24.88) |  |  |
| 2 |  |  |  | 0.022 | 0.881 |
| Community Elderly Care Services | 490 (74.24) | 340 (74.07) | 150 (74.63) |  |  |
| 1 | 170 (25.76) | 119 (25.93) | 51 (25.37) |  |  |
