# Supplementary material for: Development of a nomogram model for predicting dementia risk in the older adult population of Weifang, Shandong Province, China: based on the biopsychosocial model
Source: Front Public Health. 2025 Feb 21;13:1499820. doi: 10.3389/fpubh.2025.1499820 (PMC11885151; doi:10.3389/fpubh.2025.1499820)
Supplement: Supplementary file 3 [file Data_Sheet_3.docx]

# Load data

library(readxl)

library(foreign)

library(rms)

library(rrtable) # Output table as Word document

library(ggplot2) # Save images

library(psych)

library(caret)

library(Hmisc)

library(psych)

library(car)

setwd('C:/Users/Administrator/Desktop/DementiaYing/results/3.frontier/fixed/data_filled.xlsx')

data_filled <- read_excel("C:/Users/Administrator/Desktop/DementiaYing/results/3.frontier/fixed/data_filled.xlsx")

completed_data <- as.data.frame(data_filled)

table(completed_data$Dementia)

for (col in names(completed_data)) {

completed_data[[col]] <- as.numeric(as.factor(completed_data[[col]]))

} # Convert all variables from factors to numeric

table(completed_data$Dementia)

completed_data$Dementia <- ifelse(completed_data$Dementia == 2, 1, ifelse(completed_data$Dementia == 1, 0, completed_data$Dementia))

x1 <- as.matrix(completed_data[, -1])

y1 <- completed_data$Dementia

library(MASS)

# Random number seed

set.seed(123) # Random number generator

ind <- sample(2, nrow(completed_data), replace = TRUE, prob = c(0.7, 0.3))

# Split dataset

train1 <- completed_data[ind == 1, ] # The training data set

test1 <- completed_data[ind == 2, ]

str(test1) # Confirm it worked

str(train1) # Confirm it worked

train_x1 <- as.matrix(train1[,-1])

train_y1 <- train1$Dementia

test_x1 <- as.matrix(test1[,-1])

test_y1 <- test1$Dementia

#### Lambda minimum corresponding to the number of variables

library(glmnet)

lasso <- glmnet(train_x1, train_y1, family = "binomial", alpha = 1) # y is continuous then use gaussian

print(lasso)

#### Draw working plot

# par(lwd=2, font=2) # lwd controls line thickness, font controls font (2 means bold)

plot(lasso, xvar = "lambda", label = TRUE)

##### Compute regression coefficients

lasso.coef <- predict(lasso, s = 0.000180, type = "coefficients") # s is the lambda from above

lasso.coef

####### Cross-validation

set.seed(317)

train_y1 <- as.numeric(train_y1)

lasso.cv = cv.glmnet(train_x1, train_y1, nfolds = 10) # 10-fold

plot(lasso.cv)

lasso.cv$lambda.min # minimum, check this for this dataset

lasso.cv$lambda.1se # one standard error away

coef(lasso.cv, s = "lambda.min") # If lambda.1se is better, choose that one

############################################################# Factor Variables

completed_data$Gender <- as.factor(completed_data$性别)

completed_data$PhysicalDisability <- as.factor(completed_data$身体残疾)

completed_data$HearingAbnormality <- as.factor(completed_data$听力异常)

completed_data$Hyperlipidemia <- as.factor(completed_data$高血脂)

completed_data$HeartDisease <- as.factor(completed_data$心脏病)

completed_data$AttentionDeficit <- as.factor(completed_data$注意力不集中)

completed_data$Hope <- as.factor(completed_data$希望)

completed_data$SelfRatedHealth <- as.factor(completed_data$自评健康状况)

completed_data$SleepDuration <- as.factor(completed_data$睡眠时长)

completed_data$Napping <- as.factor(completed_data$午睡)

completed_data$EducationLevel <- as.factor(completed_data$文化程度)

completed_data$Residence <- as.factor(completed_data$居住地)

completed_data$Socialization <- as.factor(completed_data$社交)

completed_data$ModeratePhysicalActivity <- as.factor(completed_data$中度体力活动)

completed_data$LightPhysicalActivity <- as.factor(completed_data$轻度体力活动)

completed_data$HealthInsurance <- as.factor(completed_data$医疗保险)

# Ensure dependent variable "Dementia" is either numeric or factor

completed_data$Dementia <- as.factor(completed_data$Dementia)

# Fit logistic regression model

fit1 <- glm(Dementia ~ Gender + PhysicalDisability + HearingAbnormality + Hyperlipidemia + HeartDisease + AttentionDeficit + Hope +

SelfRatedHealth + SleepDuration +

Napping + EducationLevel + Residence + Socialization + ModeratePhysicalActivity + LightPhysicalActivity + HealthInsurance,

data = completed_data, family = "binomial")

fit1

# Stepwise regression based on AIC for variable selection

# fit2 <- step(fit1, direction = "backward", trace = FALSE)

# fit2

library(questionr)

# Calculate odds ratios for selected model

orresults <- odds.ratio(fit1)

# Convert odds ratio results to dataframe

ordf <- as.data.frame(orresults)

# View dataframe

print(ordf)

# Fit logistic regression model

fit3 <- glm(Dementia ~ AttentionDeficit + SelfRatedHealth

+ Napping + EducationLevel + Residence + Socialization + HealthInsurance,

data = completed_data, family = "binomial")

# Variance inflation factor (VIF)

vif.values <- vif(fit3)

# Output results

print(vif.values)

########################################################################

table(completed_data$Dementia)

dd <- datadist(completed_data)

options(datadist = 'dd')

#### Nomogram

library(rms)

# Fit logistic regression model

full <- lrm(Dementia ~ AttentionDeficit + SelfRatedHealth + Napping + EducationLevel + Residence + Socialization + HealthInsurance, data = completed_data)

# Create nomogram

nom1 <- nomogram(full, fun = plogis, fun.at = c(.001, .01, .05, seq(.1, .9, by = .1), .95, .99, .999), lp = F, funlabel = "rate")

par(mar = c(5, 8, 2, 2), family = "Times New Roman")

plot(nom1, col.grid = gray(c(0.8, 0.95)))

# Evaluation

train <- subset(train1, select = c("Dementia", "AttentionDeficit", "SelfRatedHealth", "Napping", "EducationLevel"

,"Residence", "Socialization", "HealthInsurance"))

test <- subset(test1, select = c("Dementia", "AttentionDeficit", "SelfRatedHealth", "Napping", "EducationLevel"

,"Residence", "Socialization", "HealthInsurance"))

dd <- datadist(train)

options(datadist = 'dd')

dd <- datadist(test)

options(datadist = 'dd')

train_x <- train[,-1]

train_y <- train1$Dementia

test_x <- test[,-1]

test_y <- test1$Dementia

# Convert all categorical variables to factors

train$Dementia <- as.factor(train$Dementia)

# Convert categorical variables to numeric

train$AttentionDeficit <- as.numeric(train$AttentionDeficit)

train$SelfRatedHealth <- as.numeric(train$SelfRatedHealth)

train$Napping <- as.numeric(train$Napping)

train$EducationLevel <- as.numeric(train$EducationLevel)

train$Residence <- as.numeric(train$Residence)

train$Socialization <- as.numeric(train$Socialization)

train$HealthInsurance <- as.numeric(train$HealthInsurance)

# Convert all categorical variables to factors

test$Dementia <- as.factor(test$Dementia)

# Convert categorical variables to numeric

test$AttentionDeficit <- as.numeric(test$AttentionDeficit)

test$SelfRatedHealth <- as.numeric(test$SelfRatedHealth)

test$Napping <- as.numeric(test$Napping)

test$EducationLevel <- as.numeric(test$EducationLevel)

test$Residence <- as.numeric(test$Residence)

test$Socialization <- as.numeric(test$Socialization)

test$HealthInsurance <- as.numeric(test$HealthInsurance)

## ROC

full.fit <- glm(Dementia ~ AttentionDeficit + SelfRatedHealth

+ Napping + EducationLevel + Residence + Socialization + HealthInsurance, data = train, family = "binomial")

full.fit1 <- glm(Dementia ~ AttentionDeficit + SelfRatedHealth

+ Napping + EducationLevel + Residence + Socialization + HealthInsurance, data = test, family = "binomial")

# Generate predicted probabilities

train$predicted_prob <- predict(full.fit, type = "response")

test$predicted_prob <- predict(full.fit1, type = "response")

# Calculate ROC curve for each predictor

roc_model <- roc(train$Dementia, train$predicted_prob)

roc_attention <- roc(train$Dementia, train$AttentionDeficit)

roc_health <- roc(train$Dementia, train$SelfRatedHealth)

roc_nap <- roc(train$Dementia, train$Napping)

roc_education <- roc(train$Dementia, train$EducationLevel)

roc_residence <- roc(train$Dementia, train$Residence)

roc_social <- roc(train$Dementia, train$Socialization)

roc_insurance <- roc(train$Dementia, train$HealthInsurance)

auc_value

auc_value1 <- auc(roc_model)

# Plot ROC curves

plot(roc_model, col = "black", main = "ROC Curves")

lines(roc_attention, col = "red")

lines(roc_health, col = "blue")

lines(roc_nap, col = "green")

lines(roc_education, col = "purple")

lines(roc_residence, col = "orange")

lines(roc_social, col = "pink")

lines(roc_insurance, col = "brown")

# Add light gray grid lines

grid(col = "gray70") # Light gray grid lines

# Adjust legend position and add space

legend(x = 0.55, y = 0.35, cex = 0.5, text.font = 2, legend = c(

paste("Dementia Model (AUC =", round(auc(roc_model), 3), ")"),

paste("Lack of concentration (AUC =", round(auc(roc_attention), 3), ")"),

paste("Self-assessed health status (AUC =", round(auc(roc_health), 3), ")"),

paste("Napping (AUC =", round(auc(roc_nap), 3), ")"),

paste("Educational level (AUC =", round(auc(roc_education), 3), ")"),

paste("Residence (AUC =", round(auc(roc_residence), 3), ")"),

paste("Socialization (AUC =", round(auc(roc_social), 3), ")"),

paste("Medical Insurance (AUC =", round(auc(roc_insurance), 3), ")")

),

col = c("black", "red", "blue", "green", "purple", "orange", "pink", "brown"),

lwd = 2, inset = c(0.005, 0.005), bty = "n" # bty="n" removes the box, inset adjusts distance

)

# Calculate ROC curve for test data

roc_model <- roc(test$Dementia, test$predicted_prob)

roc_attention <- roc(test$Dementia, test$AttentionDeficit)

roc_health <- roc(test$Dementia, test$SelfRatedHealth)

roc_nap <- roc(test$Dementia, test$Napping)

roc_education <- roc(test$Dementia, test$EducationLevel)

roc_residence <- roc(test$Dementia, test$Residence)

roc_social <- roc(test$Dementia, test$Socialization)

roc_insurance <- roc(test$Dementia, test$HealthInsurance)

auc_value2 <- auc(roc_model)

# Plot ROC curves

plot(roc_model, col = "black", main = "ROC Curves")

lines(roc_attention, col = "red")

lines(roc_health, col = "blue")

lines(roc_nap, col = "green")

lines(roc_education, col = "purple")

lines(roc_residence, col = "orange")

lines(roc_social, col = "pink")

lines(roc_insurance, col = "brown")

# Add light gray grid lines

grid(col = "gray70") # Light gray grid lines

# Adjust legend position and add space

legend(x = 0.55, y = 0.35, cex = 0.5, text.font = 2, legend = c(

paste("Dementia Model (AUC =", round(auc(roc_model), 3), ")"),

paste("Lack of concentration (AUC =", round(auc(roc_attention), 3), ")"),

paste("Self-assessed health status (AUC =", round(auc(roc_health), 3), ")"),

paste("Napping (AUC =", round(auc(roc_nap), 3), ")"),

paste("Educational level (AUC =", round(auc(roc_education), 3), ")"),

paste("Residence (AUC =", round(auc(roc_residence), 3), ")"),

paste("Socialization (AUC =", round(auc(roc_social), 3), ")"),

paste("Medical Insurance (AUC =", round(auc(roc_insurance), 3), ")")

),

col = c("black", "red", "blue", "green", "purple", "orange", "pink", "brown"),

lwd = 2, inset = c(0.005, 0.005), bty = "n" # bty="n" removes the box, inset adjusts distance

)

# Calibration curve

c_train <- lrm(Dementia ~ AttentionDeficit + SelfRatedHealth

+ Napping + EducationLevel + Residence + Socialization + HealthInsurance, data = train, x = T, y = T)

c_test <- lrm(Dementia ~ AttentionDeficit + SelfRatedHealth

+ Napping + EducationLevel + Residence + Socialization + HealthInsurance, data = test, x = T, y = T)

# Use calibrate() to draw calibration curve

cal1 <- calibrate(c_train, method = "boot", B = 1000) # B is the number of bootstraps

plot(cal1, main = "Calibration Curve")

cal2 <- calibrate(c_test, method = "boot", B = 1000) # B is the number of bootstraps

plot(cal2, main = "Calibration Curve")

# Decision Curve Analysis (DCA)

library(rmda)

train$Dementia <- as.numeric(train$Dementia) - 1 # Convert "No" to 0, "Yes" to 1

# Refit the model

model1 <- decision_curve(Dementia ~ AttentionDeficit + SelfRatedHealth +

Napping + EducationLevel + Residence + Socialization + HealthInsurance,

family = binomial(link = 'logit'),

data = train,

thresholds = seq(0, .8, by = .05),

bootstraps = 10)

# Plot DCA curve

plot_decision_curve(model1,

curve.names = "Model",

col = c("red"),

confidence.intervals = F # Do not show confidence intervals

)

test$Dementia <- as.numeric(test$Dementia) - 1 # Convert "No" to 0, "Yes" to 1

# Refit the model

model2 <- decision_curve(Dementia ~ AttentionDeficit + SelfRatedHealth +

Napping + EducationLevel + Residence + Socialization + HealthInsurance,

family = binomial(link = 'logit'),

data = test,

thresholds = seq(0, .8, by = .05),

bootstraps = 10)

# Plot DCA curve

plot_decision_curve(model2,

curve.names = "Model",

col = c("red"),

confidence.intervals = F # Do not show confidence intervals

)
